# Rush Medical College—Spring Course of Lectures

**Published:** 1868-07-01

**Authors:** 


### RUSH MEDICAL COLLEGE. — SPRING COURSE OF LECTURES.

Prof. Allen: — Allow us, in behalf of the class in attendance at the Spring Course of Lectures at Rush Medical College, to express, through the columns of the Journal: first, our high estimate of the opportunities for study so generously afforded medical students, by the faculty of the above-named institution; second, our thorough appreciation of the course of Lectures delivered by Dr. C. T. Parkes, on Comparative and Descriptive Anatomy; Prof. Blaney and Dr. I. N. Danforth, on Toxicology and Medical Jurisprudence; Prof. Freer, on Physiology and Microscopic Anatomy; Dr. Fenn, on Obstetrics and Diseases of Women and Children; Dr. J. E. Owen, on Surgery; and Dr. Wm. C. Lyman, on Surgery and Diseases of the Chest. We have also witnessed, with much pleasure and profit, Prof. Gunn's numerous and very successful operations at the weekly surgical Clinic; Prof. Powell's vivisections, illustrating the course on Toxicology; and Dr. W. R. Marsh's careful examination of cases presented at the medical Clinic. We are also under obligations for opportunities for extended observation at the County and City Hospital, directed, in the surgical department by Dr. R. G. Bogue, and in the medical by Dr. Thomas Bevan; and for similar favors at the Eye and Ear Infirmary, under the direction of Prof. E. L. Holmes.

These gentlemen have labored with unmistakable interest in behalf of the class, leaving no available means unused to make the Spring Course of Lectures, just closed, pleasant and profitable; and we hereby tender them, each and all, our thanks, with assurances of high personal esteem.

During the Spring Term, Prof. Blaney has conducted, most successfully, a course of instruction in Practical Chemistry, affording a rare opportunity for the acquisition of most useful knowledge — the possession of which demonstrates to every student the beauties of chemical science, together with its positive character, which can only be fully comprehended after practical manipulations in the laboratory. A large proportion of the class have attended this course, and desire us to say that they are a unit in their hearty appreciation of the benefits thus derived, and also in their appreciation of Prof. Blaney as a teacher of chemistry.

All things considered, we deem this Spring Course of great value to us as medical students, furnishing opportunities for observation and instruction not attainable in the more formal regular course; and we shall ever be thankful for its thoroughly taught lessons and pleasant associations.

F. L. Wadsworth,

M. Donnelly, } *Committee.*

R. Broughton,

Chicago, Ill., *May* 30*th*, 1868.

